# “Infiltrative” Versus “Mass-Forming” Pancreatic Cancer: A New Radiological Classification System for Pancreatic Head Ductal Carcinoma and Its Pathological Correlation

**DOI:** 10.1089/pancan.2022.0003

**Published:** 2022-10-05

**Authors:** Muhammad B. Darwish, Shankar Logarajah, Patrick J. McLaren, Annie L. Benzie, Jason Schmidt, Assad J. Saad, Mauricio Salicru, Terence Jackson, Shyam Vedantam, Jeffery Carenza, Clayton Sanders, Kei Nagatomo, Edward Cho, Houssam Osman, Dhiresh Rohan Jeyarajah

**Affiliations:** ^1^ Department of Surgery, Methodist Richardson Medical Center, Richardson, Texas, USA.; ^2^ Department of Pathology, Methodist Health System, Dallas, Texas, USA.; ^3^ Department of Surgery, Akron General Hospital, Akron, Ohio, USA.; ^4^ Department of Internal Medicine, Jackson Memorial Hospital, Miami, Florida, USA.; ^5^ Texas Radiology Associates, Plano, Texas, USA.; ^6^ Department of Surgery, Morehouse General Hospital, Bastrop, Louisiana, USA.; ^7^ Department of Surgery, The University of Oklahoma at Tulsa, Tulsa, Oklahoma, USA.; ^8^ Department of Surgery, TCU/UNTHSC School of Medicine, Fort Worth, Texas, USA.

**Keywords:** mass forming, infiltrative, pancreas cancer, pancreatic ductal adenocarcinoma, resection margin

## Abstract

**Purpose:**

Resectability in localized pancreatic ductal adenocarcinoma (PDAC) is deemed through radiological criteria. Despite initial evaluation classifying tumors as “resectable,” they often have ill-defined borders that can result in more extensive cancer than predicted on final pathology analysis. We attempt to categorize these tumors radiologically and define them as “infiltrative” and contrast them to more well-defined or “mass-forming” tumors and assess their correlation with surgical oncological outcomes. We hypothesize that mass-forming lesions will result in fewer positive resection margins.

**Methods:**

Patients diagnosed with PDAC of the head of the pancreas and who underwent subsequent curative intent resection between 2016 and 2018 were included. A retrospective chart review of patients was conducted and computed tomography images at the time of diagnosis were reviewed by two radiologists and scored as “mass forming” or “infiltrative” using a newly developed classification system. These classifications were then correlated with margin status.

**Results:**

Sixty-eight consecutive pancreatoduodenectomies performed for PDAC from 2016 to 2018 were identified. After screening, 54 patients were eligible for inclusion. Radiologically defined mass-forming lesions had a trend toward a lower rate of positive resection margins (35.7% vs. 50.0%; *p* = 0.18), specifically the bile duct margin and pancreas margin as well as an overall larger size (4.03 cm vs. 3.25 cm, *p* = 0.02) compared with infiltrative lesions.

**Conclusion:**

We propose a new radiological definition of PDAC into “mass forming” and “infiltrative,” a nomenclature that resonates with other tumor sites. Infiltrative lesions trended toward a higher rate of positive resection margins. This classification may help tailor therapy for infiltrative tumors toward a neoadjuvant approach even if they appear resectable.

## Introduction

Pancreatic ductal adenocarcinoma (PDAC) is one of the leading causes of cancer morbidity and mortality in the United States with an extremely poor prognosis.^1^ It is estimated that 60,430 people will be diagnosed with pancreatic cancer in 2021 and 48,220 will die of it.^1^ The estimated 5-year survival rate of PDAC is ∼10%, a notable improvement from 5.3% 20 years ago.^2^ Despite continuous developments in better detection, safer surgery, and treatment, pancreatic adenocarcinoma remains difficult to treat with 5-year survival rates well below the most common cancers in the United States. Improvements in detection, preoperative workup, and treatment are desperately needed.

Surgery is the only potentially curative option for patients diagnosed with PDAC with resection margin status being one of the most important pathological predictive factors for overall and recurrence-free survival.^3–5^ The importance of achieving true negative resection margins has prompted efforts to standardize tumor sampling and pathology reporting to reduce the risk of reporting incompletely assessed margins as negative.^6,7^ Despite advances in imaging, PDACs tend to be understaged preoperatively that can result in positive resection margins, even when the lesion appears to be resectable.^8,9^

We have found that some PDACs are inherently more “infiltrative” when compared with other “mass-forming” lesions despite being deemed resectable on preoperative imaging. Current radiological classification of resectability of PDAC relies on the involvement, or lack thereof, of the surrounding vessels but does not factor in the overall morphology of the tumor. In discussions with our body imagers, the finding of termination of the bile duct and/or pancreatic duct without a discrete mass on CT or magnetic resonance imaging (MRI) would lead them to “imply” a mass, rather than actually identify a discrete lesion.

In contrast, there are lesions that enhance differentially from normal pancreas and take on a “mass-like” appearance. We have coined the former as “infiltrative” and the latter as “mass forming.” This resonates with the nomenclature in hepatocellular carcinoma (HCC).^10^ The purpose of this study is twofold: (1) to define imaging criteria for “mass-forming” and “infiltrative” pancreatic adenocarcinoma and (2) to assess the correlation of these two entities with pathological margin status. We hypothesize that mass-forming lesions will correlate with fewer positive resection margins.

## Methods

This study was approved by our institution's IRB. A retrospective chart review was undertaken of consecutive pancreatoduodenectomies performed for PDACs from August 2016 to October 2018. Patient demographics, comorbid conditions, and postoperative pathological information including tumor staging, lymph node staging, and resection margin status were collected and analyzed. Imaging obtained during initial workup was retrieved and independently reviewed in consensus conference by two body-imaging fellowship-trained radiologists. Radiological criterion for classifying tumors as “mass forming” versus “infiltrative” was established and is described in Table 1.

**Table 1.** Summary of the Proposed Pancreatic Adenocarcinoma Classification Criteria

Feature	Mass forming	Infiltrative
Radiological		
Border	Presence of a well-defined interface	Absence of a well-defined interface
Mass	Visible and clearly identifiable lesion	Inferred in the absence of a clear lesion with presence of pancreatic and/or common bile ductal dilation

**FIG. 1.**
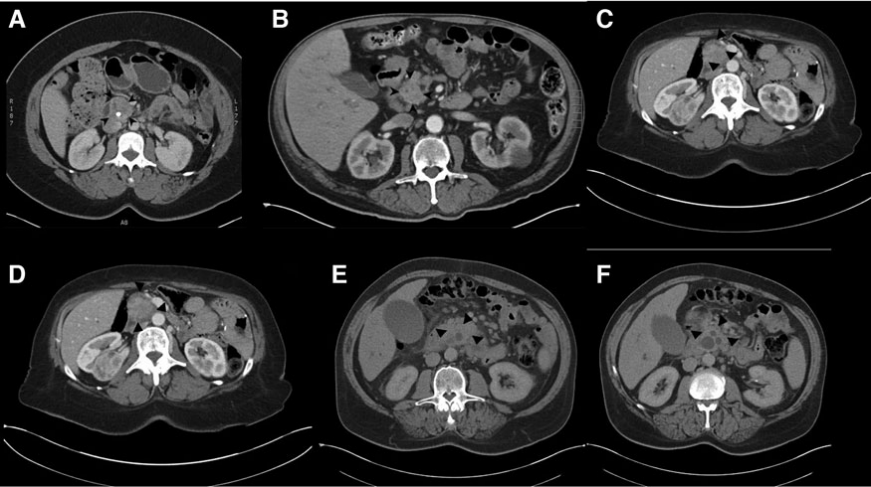
Representative axial slice images of lesions characterized as mass forming **(A–C)** and infiltrative **(D–F)**. Lesions are circumscribed within the image by black arrow heads.

Radiological classification was based on the presence or absence of a well-defined interface between the primary lesion and surrounding tissue (Fig. 1). The lack of a mass but inferred lesion due to termination of the pancreatic and/or bile duct and lack of interface between the tumor and surrounding tissue were used to designate a lesion as “infiltrative.” Two experienced body radiologists were blinded to the patients' clinical and pathological information and reviewed imaging in consensus conference. All imaging studies reviewed were triple phase pancreas protocol CT scans.

Cases were stratified into groups by their radiological classification of “mass forming” or “infiltrative” and correlated with final margins on resected pathological specimen. Our institutional protocol for PD specimens involves a five-color inking along the following areas:^7^ (1) portal vein (PV) groove, (2) superior mesenteric artery (SMA) margin, (3) retroperitoneal margin, (4) pancreas neck transection margin, and (5) bile duct margin.

A positive margin was defined by pathologists by the presence of tumor cells on microscopic evaluation of each margin, a single positive margin out of the five examined would lead to a designation of a positive margin status of the whole tumor. Univariate analysis utilizing the chi-square test, Student's *t*-test, and Mann–Whitney *U* tests was performed. A *p*-value of <0.05 was considered statistically significant.

## Results

A total of 68 pancreatoduodenectomies were retrospectively reviewed. Three patients were excluded due to incomplete data and 11 patients were excluded due to receiving neoadjuvant therapy (NAT), leaving 54 patients eligible for inclusion. Thirty-two patients were women (59.3%) and 22 were men (40.7%) with an average age of 66.9 years old (range 49–84). All study patients underwent primary resection of their tumors. Most patients were pT3 (40/54; 74.1%) and pN1 (28/54; 51.9%) with a total of 24 patients with a positive resection margin (24/54; 44.4%).

A margin was considered positive when there was tumor on ink. The most commonly positive margin on resection was the PV margin (13/24; 54.2%) followed by the SMA margin (9/24; 37.5%), and pancreatic margin (6/24; 25.0%). This high rate of R1 positivity is impacted by our five-color inking of the specimen with thorough sampling of the PV and SMA margins.^7^

**FIG. 2.**
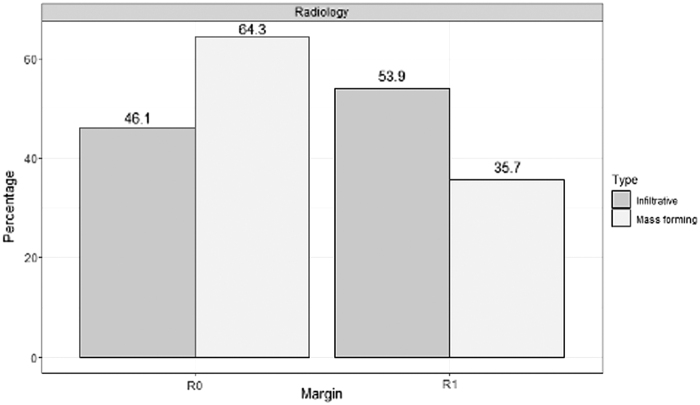
Rates of margin positivity of infiltrative and mass-forming lesions on pathological and radiological classification.

Twenty-eight cases (51.9%) were classified as mass forming and 26 (48.1%) as infiltrative lesions (Fig. 2). Infiltrative lesions were found to have a statistically significant smaller mean tumor size (3.25 ± 1.23 cm, *n* = 26) than mass-forming lesions (4.03 ± 1.14 cm, *p* = 0.02). Infiltrative lesions were noted to have a higher overall resection margin positivity (53.9% vs. 35.7%; *p* = 0.18) with the most commonly positive resection margin being the PV margin (7/14; 50.0%) (Table 2). There was no statistically significant difference in the median number of positive lymph nodes (*p* = 0.43) or tumor grade (*p* = 0.25) (Table 2).

**Table 2.** Summary of the Pathological Data of Infiltrative Versus Mass-Forming Lesions Based on Radiological Classification

	Radiology		
Variable	Mass forming (n = 28)	Infiltrative (n = 26)	*p*
Mean tumor size (cm)	4.03 ± 1.14	3.25 ± 1.23	**0.02**
Median positive LN	2.50 (4.50)	4.00 (5.00)	0.43
Median total LN examined (IQR)	27.50 (13.50)	27.00 (12.25)	0.98
Median tumor grade	2.50 ± 0.58	2.00 ± 0.69	0.25
Superior pancreatic LN (positive)	3 (10.7%)	3 (11.5%)	0.92
Lateral CBD LN (positive)	0 (0.0%)	2 (7.7%)	0.15
Resection margin (positive)	10 (35.7%)	14 (53.9%)	0.18
Portal vein margin (positive)	6 (21.4%)	7 (26.9%)	0.64
Bile duct margin (positive)	0 (0.0%)	3 (11.5%)	0.06
SMA margin (positive)	5 (17.9%)	4 (15.4%)	0.81
Pancreas margin (positive)	1 (3.6%)	5 (19.2%)	0.07
Perineural invasion	25 (89.3%)	20 (76.9%)	0.22
Lymphovascular invasion	19 (67.9%)	13 (50.0%)	0.18
Tumor grade			0.25
1	1 (3.6%)	4 (15.4%)	
2	13 (46.4%)	13 (50.0%)	
3	14 (50.0%)	9 (34.6%)	
pT			0.63
1	0 (0.0%)	1 (3.8%)	
2	7 (25.0%)	4 (15.4%)	
3	20 (71.4%)	20 (76.9%)	
3b	1 (3.6%)	1 (3.8%)	
pN			0.62
0	11 (39.3%)	7 (26.9%)	
1	13 (46.4%)	15 (57.7%)	
2	4 (14.3%)	4 (15.4%)	

Bold values emphasize statistical significance.

CBD, common bile duct; IQR, interquartile range; LN, lymph node; pN, pathological node stage; pT, pathological tumor stage; SMA, superior mesenteric artery.

## Discussion

We propose a new radiological classification of PDACs of the head of the pancreas based on defined characteristic as either “infiltrative” or “mass forming.” This study found infiltrative PDAC lesions to have a higher rate of positive resection margins compared with mass-forming lesions and an overall smaller tumor size. In cases with positive margins, the PV margin was the most commonly positive margin with a trend toward a significant difference in bile duct margin positivity.

This study also illustrates the current underestimation of margin status by imaging as shown by the high rate of positive resection margins. The difference in size between infiltrative and mass-forming lesions may be explained by the fact that cases with pancreatic and/or common bile duct dilation on imaging were considered infiltrative even in the absence of an identifiable mass.

Further characterization of tumor biology between these two types of lesions needs to be assessed to determine whether NAT may play a role given the higher propensity for margin positivity in infiltrative tumor types. However, radiographic classification of lesions as mass forming or infiltrative did not correlate with final pathological findings, which highlights the need for improved methods of evaluating PDACs in the preoperative setting. This may be partly due to the fact that PDACs that appear like a “mass” on gross pathological examination may still have “infiltrative” borders on microscopic examination.

The gold standard imaging modality for PDACs is multidetector CT (MDCT) with IV contrast pancreatic protocol.^11^ MDCT pancreatic protocol is characterized by thinner sequential sections measuring 0.5–1 mm, a pancreatic parenchymal acquisition phase occurring 40–50 sec after IV contrast injection, and a portal venous phase 65–70 sec after contrast injection.^11^

MRI of the abdomen without and with IV contrast is reported to be similar in sensitivity and specificity and can be used interchangeably.^12^ Current morphological parameters used in the evaluation of PDACs on imaging include attenuation (hypo-, iso-, hyper-attenuation), size, location, pancreatic duct narrowing (with or without dilation), and biliary tree abrupt cutoff (with or without upstream dilation).^11^

Vascular evaluation relies on the degree of encasement in the presence of contact between the tumor and surrounding vessels, including the hepatic artery, SMA, celiac axis, and PV/superior mesenteric vein (SMV) with different characterizations of resectability for each.^13,14^ This classification assumes no deformity of the involved vessel. Some authors argue that the presence of vascular involvement indicates invasion regardless of the degree of encasement, although there is not a consensus regarding this point among experts in the field.^11^

Current imaging criterion does not include a dedicated parameter to the overall appearance of the tumor on imaging. The criteria proposed in this study can be likened to the imaging criteria used for classifying HCC as mass versus infiltrative, where mass is well defined and infiltrative is poorly defined on imaging.^15^ The difference in the case of PDAC is the evaluation of the presence or absence of a well-defined interface between the primary lesion and surrounding tissue.

The importance of achieving a negative resection margin in PDAC surgery is well established in the literature.^4,6,16–18^ In 2019, the results of the European Study Group trial showed a significant improvement in overall and recurrence-free survival in patients with negative resection margins and an increased risk for local recurrence in patients with positive resection margins.^4^ In addition, patients with more than one positive margin had significantly reduced survival compared with a single positive margin.^4^ The importance of achieving an R0 resection has prompted efforts into standardizing the pathology reporting of PDAC.^6^

Verbeke et al proposed a new standard inking scheme of the Whipple specimen that specifically emphasizes the SMV groove margin.^6^ This has resulted in an overall increase in positive resection margins that the authors attribute to increased sensitivity.^6^ The data would suggest that using this more stringent pathological evaluation results in a higher positive resection margin rate, but this means that all negative margins are most likely true negative resections. This translates into a higher survival for the true negative resection when not contaminated by false negatives.^6^

Current National Comprehensive Cancer Network (NCCN) guidelines specify management pathways based on the resectability of the primary tumor and the presence or absence of metastasis.^19^ Patients with resectable disease have the option of undergoing upfront surgery without NAT, but can still be considered in high-risk circumstances.^19^ The classification proposed in this study may offer patients with otherwise resectable disease but “infiltrative” appearance on imaging the option of NAT that would have otherwise not been considered. Given that results of this study demonstrated increased positive margins in “infiltrative” lesions, this approach may provide the means to improving outcomes in carefully selected patients.

Limitations of this study include that it is a single-institution retrospective analysis with a limited patient cohort and lack of survival data. In addition, the criterion defined for this study is subjective in nature, which presents a challenge in reproducing the results of this study but provides a basis for further study to define objective criteria. Further research with a larger sample size focused on defining classification criteria in more objective terms is required.

## Conclusion

We propose a new radiological definition of pancreatic head cancers into “mass forming” and “infiltrative,” a nomenclature that resonates with other tumor sites. Infiltrative lesions trended toward a higher rate of positive resection margins, especially the bile duct margin, and a smaller overall size. This classification may help tailor these infiltrative tumors toward a neoadjuvant approach even if they appear clearly resectable.

## Abbreviations Used

CBD: common bile duct
CT: computed tomography
IQR: interquartile range
LN: lymph node
MDCT: multidetector CT
MRI: magnetic resonance imaging
NAT: neoadjuvant therapy
PDAC: pancreatic ductal adenocarcinoma
pN: pathological node stage
pT: pathological tumor stage
PV: portal vein
SMA: superior mesenteric artery
SMV: superior mesenteric vein
